# Prevalence and correlates of male partner involvement in antenatal care services in eastern Kenya: a cross-sectional study

**DOI:** 10.11604/pamj.2022.41.167.31535

**Published:** 2022-02-28

**Authors:** Pascalyne Kavesa Nyamai, Joseph Matheri, Kenneth Ngure

**Affiliations:** 1 Jomo Kenyatta University of Agriculture and Technology, Nairobi, Kenya

**Keywords:** Male partner, antenatal care, maternal health, physical support, financial support, decision-making support

## Abstract

**Introduction:**

male partner involvement in antenatal care (ANC) contributes to improved maternal health outcomes, but has been wanting in sub-Saharan Africa. We investigated the prevalence and factors associated with male involvement in ANC.

**Methods:**

this was a cross-sectional survey conducted in November and December 2019 in Kitui East sub-county, Kenya. We recruited men above 18 years whose spouses had given birth 12-months prior to the study. Data were collected at the household level using an interviewer-administered questionnaire. Male involvement was defined as provision of physical, psycho-social, decision-making, and financial support, which was measured through twelve questions. Factor scores of the twelve questions were generated by fitting a Rasch model. Participants who scored at least 75% were involved. Bivariate and multivariate logistic regression models were fitted to identify the independent predictors of male involvement.

**Results:**

a total of 300 participants were interviewed. The mean age was 36.7 years (SD=±7.6), 52.3% had primary level education, 64.3% had between 1-3 children, 44.6% were 5 years older than their spouses, while 37.3% earned between $50-$100 per month. The prevalence of male involvement in ANC was 61% (95%C.I: 55.7%, 66.3%) and was positively associated with previous ANC attendance by the spouse (AOR= 4.96, 95% CI: 2.37, 10.38, p<0.001), having 1-2 and 3-4 children (AOR= 4.57, 95% CI: 1.70, 12.31, p=0.003 and AOR= 4.84, 95% CI: 1.59, 14.79, p=0.006) respectively. On the contrary, participants who lacked knowledge on the minimum ANC visits (AOR= 0.37, 95% CI: 0.17, 0.83, p=0.016), unplanned pregnancy (AOR=0.22, 95% CI: 0.10, 0.48, p<0.001), and individual financial decision-making (AOR= 0.42, 95% CI: 0.21, 0.89, p=0.023) were less likely to be involved.

**Conclusion:**

more than half of the participants reported involvement in ANC, which was significantly associated with previous ANC experience and having less than four children. Empowering men with knowledge on ANC and joint decision-making with their spouses is imperative in order to improve male involvement.

## Introduction

Despite a significant global decline in maternal deaths over the past two decades, the rate of maternal mortality remains unacceptably high [1]. Low and middle income countries account for approximately 94% of these maternal deaths annually, with about two thirds occurring in sub-Saharan Africa (SSA) [2]. The estimated maternal mortality ratio (MMR) of SSA is 542 deaths per 100,000 live births, which is more than double the global maternal mortality ratio of 211 deaths per 100,000 live births [2]. In Kenya, the 2014 demographic and health survey reported a MMR of 362 deaths per 100,000 live births, with large regional disparities [3]. Counties such as Mandera, Wajir and Turkana have the highest burden and reported relatively high MMR of 3795, 1683 and 1594, respectively [4].

Most of these maternal deaths which occur as a result of complications during and following pregnancy and childbirth [5-8] are preventable through timely provision of adequate and skilled maternal health care before, during and after childbirth [9,10]. Antenatal care has been documented as a high impact intervention in reducing maternal mortality [11]. Antenatal care (ANC) is defined as the care provided by skilled healthcare professionals to pregnant women and adolescent girls to ensure the best health conditions for both mother and baby during pregnancy [12]. While four ANC visits are the recommended minimum, fewer women (52%) especially in SSA attain this score [13,14]. In Kenya, only 58% of women attend the four recommended ANC visits during pregnancy [3]. Despite the wide availability of ANC services in many settings, women are constrained from accessing the services by a variety of factors including long distance to health facilities, lack of information, inadequate and poor quality services and cultural beliefs and practices [15-18].

Previous research has established that male partner involvement in ANC is positively associated with their pregnant partners´ utilization of reproductive health care services, thus improving maternal and child health outcomes [19]. Unfortunately, over the years, male partner involvement in antenatal care has been wanting globally and more so in low and middle income countries. In particular, countries in SSA that account for the larger share of maternal deaths have reported correspondingly low levels of male involvement which include 54% in Tanzania [20], 29.8% in Ethiopia [21], 6% in Wakiso District, Uganda [22] and 26% in Nairobi, Kenya [23].

Although male partner support is of critical importance, there is a paucity of evidence on factors influencing their involvement during ANC in Kenya. Previous studies have mainly examined the level, barriers and effect of male involvement on utilization of ANC services [23-25]. Other studies have documented individual domains of male involvement such as financial, physical and decision-making support, and associated factors [26-29]. To our knowledge, there is a dearth of empirical evidence on how the four main domains (physical, financial, psycho-social and decision-making support) interact to influence male involvement in ANC. The objective of this study was to establish the proportion of male partners involved in ANC, and identify the factors influencing male partner involvement in ANC in Kitui East sub-County, Kenya.

## Methods

**Study design and study setting:** this study was a descriptive cross-sectional survey that was conducted in November and December 2019, in Kitui East sub-county. Kitui East is one of the eight sub-counties in Kitui County that is situated in southeastern Kenya. The sub-county has approximately 17,143 households and has an estimated population of 123,239 individuals. The main economic activity is farming although it is beset by sporadic rainfall. Kitui East sub-County was selected because it is one of the regions that continue to record high rates of maternal mortality, low rates of ANC and skilled birth attendance, and that limited studies have addressed this phenomenon to date [3].

**Study participants:** eligible study participants were men aged 18 years and above, whose female partners had given birth 12 months prior to the study. We derived a sample size of 297 participants using Cochran´s formula [30] with 5% precision at a 95% confidence interval, and estimated the proportion of male partner involvement in ANC using a similar study by Aluisio [23], which documented 26.2% male involvement. The sampling proportion was derived from the 2014 Kenya Demographic and Health Survey [3], where there were 438 live births from 850 households in the five year period. This translated to approximately 88 births per year, and about one reported birth per 10 households. Using this sampling proportion, we estimated that we needed to visit 2,970 households out of the approximately 17,143 households in Kitui East sub-County, in order to recruit a sample of 297 participants.

Participants were recruited using multi-stage purposive and random sampling. In the first stage, 3 out of the 6 wards in Kitui East sub-county were purposively selected to ensure geographic spread and representation of the rural and peri-urban population. The 2,970 households were then equally distributed across the 3 wards translating to 990 households per ward. Then at ward level, we identified all the community health units and randomly selected 990 households from the household registers. Community health workers from community units covering the selected households were engaged to help identify households with potential participants using the eligibility criteria. The community health workers then guided the study team to the selected households. At the household level, the research assistants screened the potential participants for eligibility using a recruitment script, then invited eligible participants to the study. Those who expressed interest were then consented by the research assistants. Where potential participants were unavailable, or the participants were ineligible or not interested in participation, a replacement household was visited until the sample size of 297 participants was achieved.

**Study variables and measurement:** data were collected using a standardized questionnaire administered by trained research assistants. Male involvement in antenatal care was measured through twelve (12) questions that directly assessed the four major domains of male involvement; physical support, psycho-social support, decision-making support and financial support. The specific questions were; *(i) “Did you ever accompany your wife to the hospital for their routine antenatal care during the most recent pregnancy?”, (ii) “During the most recent pregnancy, did you support the use of ANC by your spouse?”,(iii) “Did you encourage your wife to attend the antenatal clinic?”, (iv) “Did you approve your wife going to the ANC clinic?”, (v) “Did you ensure good nutrition for your wife?”, (vi) “Did you identify a mode of transportation to the health facility?”, (vii) “During the most recent pregnancy, did you discuss ANC with your wife?”, (viii) “Who made decision to attend ANC”, (ix) “Who decided the place of delivery?” (x) “During the most recent pregnancy, did you provide money for clinic costs and medication related to the antenatal clinic visit?” (xi) “During the most recent pregnancy, did you provide money for transport to the clinic?” (xii) “Did you save any money for emergencies related to the pregnancy?”* The respondents answered either “yes” or “no” to all questions except questions viii and ix where if either the man or both partners made the decision, it was coded as 1 or else it was coded as 0. For all other questions “Yes” was coded as 1 “No” as 0. Other variables that were assessed through a standardized questionnaire included knowledge of ANC, sources of ANC information, and perceived health provider´s attitude towards the participants. The questionnaire was pre-tested by trained research assistants who administered it among 15 participants who met the study´s eligibility criteria and were drawn from a separate ward that was not sampled for the study.

**Statistical analysis:** the completed questionnaires were first reviewed for any inconsistencies. Data were then entered into the statistical package for social sciences (SPSS) version 24, where further cleaning was done to ensure the completeness of the dataset.

**Male involvement:** data reduction techniques were used to summarize the observed male involvement variables into a few dimensions through latent variable modelling using the “eRm” [31], “ltm” [32], and “difR” [33] R package. Component internal consistency and reliability used to calculate male involvement scores were assessed by calculating Cronbach´s alpha (a), which was found to be 0.913 (95% C.I, 0.896-0.926).

Pairwise associations between the 12 males involvement variables were computed using spearman´s correlation and all variables were found to be significantly positively correlated to each other, hence all items were retained for further analysis. Factor scores were then generated by fitting a one-parameter logistic regression model, also known as the Rasch model [34]. The scores had a bimodal negatively skewed distribution, suggesting that there were two groups. Participants scoring less than zero were classified as “not-involved” while those with more than zero were classified as “involved”. Each participant needed to score a 1 in at least 9 of the 12 items (75% and above) to be considered involved.

**Determinants of male involvement:** descriptive statistics were employed to estimate the frequencies of participants´ socio-demographic characteristics and other factors that included knowledge of ANC, sources of ANC information, and perceived health provider´s attitude. The association between male involvement and other variables were estimated using bivariate logistic regression, which was first fitted to identify potential predictors and confounding factors. Variables with a p-value <0.25 were fitted into a multivariate binary logistic regression model to identify independent predictors of male involvement. Adjusted odds ratio with its 95% confidence interval was calculated to report the strength and significance of the association. All tests were two-sided and statistical significance was set at p ≤ 0.05.

**Ethical considerations:** ethical considerations were complied with before, during and after the data collection activity. Ethical approval was obtained from the Jomo Kenyatta University of Agriculture and Technology (JKUAT) Institutional Ethics Review Committee (IERC), and a research permit was obtained from the National Commission for Science, Technology and Innovation (NACOSTI). All research assistants undertook human subject´s protection training. All participants provided informed written consent and the survey interviews were conducted in locations that guaranteed both verbal and visual privacy for the participants. Participant names were not captured in the data collection tools and all completed tools were stored in locked cabinets only accessible by the principal investigator. Personal identifiers were kept separate from the data collection tools and were all destroyed upon completion of the data collection.

## Results

A total of 300 participants were enrolled in the study. The mean age of the participants was 36.7 years (SD=±7.6), and the majority (43.3%) were aged between 35-44 years. The mean age for the participants´ spouses was 30.8 years (SD=±6.1). In addition,44.6% of the participants were aged 5 years or older than their spouses. More than half (52.3%) of the respondents had primary education and below, 30.7% had secondary education and 17% had tertiary/post-secondary education. The majority (37.3%) earned between $50 - $100 per month and most of the respondents (64.3%) had between 1-3 children.

About two thirds (66.7%) of the participants had no knowledge of the required minimum number of ANC visits and 73.0% were unaware of danger signs of pregnancy. Most (73.7%) had planned for their spouse´s pregnancies, while 66% of the respondents indicated that their spouses went for ANC during previous pregnancies. With regard to decision-making on finances, 45.7% of the respondents indicated that they jointly made decisions with their spouse, 38% made the decision alone, and 16.3% indicated that their spouses made the decision. Slightly more than half (50.7%) of the respondents received information on ANC from health care providers, followed by 21.7% who received information from their spouses, and another 15.7% from mass media sources. More than half of the respondents (58.3%) indicated that the health service providers had a positive attitude, while 30% reported a negative attitude, and 11.7% had not visited the health facility (Table 1).

**Table 1:** Socio-demographic and other characteristics of the respondents.

Variable	Category	Frequency (N=300)	Percentage (%)
**Age**	18-24 Years	17	5.7
	25-34 Years	101	33.7
	35-44 Years	130	43.3
	45+	52	17.3
**Age difference**	Younger than the wife	15	5.1
	Same age	33	11.1
	1-5 Years older	116	39.2
	5+ Years Older	132	44.6
	N/A	4	
**Education Level**	Primary and Below	157	52.3
	Secondary	92	30.7
	Tertiary/ Post-secondary	51	17.0
**Spouse Education Level**	Primary and Below	183	61.0
	Secondary	74	24.7
	Tertiary/ Post-secondary	43	14.3
**Currently Living with Wife**	Yes	264	88.0
	No	36	12.0
**Level of income**	Below 5,000	94	31.3
	5,000 - 10,000	112	37.3
	11,000 - 20,000	41	13.7
	21,000 - 30,000	19	6.3
	31,000 and Above	34	11.3
**Decision on Money earned**	Respondent	114	38.0
	Wife	49	16.3
	Respondent and wife	137	45.7
**Number of children**	1-3	193	64.3
	3-4	57	19.0
	5 and Above	50	16.7
**Knowledge of the minimum required number of ANC Visits**	No	200	66.7
	Yes	100	33.3
**Aware of danger signs of pregnancy**	No	220	73.3
	Yes	80	26.7
**Pregnancy Planned**	No	79	26.3
	Yes	221	73.7
**Went to ANC during previous pregnancies**	Yes	198	66.0
	No	102	34.0
**Source of information about ANC**	Mass/Print Media	47	15.7
	Partner/Wife	65	21.7
	Health care Providers	152	50.7
	Discussion with people	36	12.0
**Provider Attitude**	Positive	175	58.3
	Negative	90	30.0
	Not applicable	35	11.7

The overall prevalence of male involvement in antenatal care was 61% (n=183), (95% C.I: 55.7%, 66.3%). However, involvement in each of the twelve male involvement variables ranged from 23.3% to 91.0%, with the majority (91%) ensuring that their spouses had good nutrition while the least (23.3%) accompanied their wife/spouse to the hospital for routine antenatal care (Table 2).

**Table 2:** male involvement in antenatal care across 12 dimensions

Male Involvement Items	n(%)
Did you ever accompany your wife to the hospital for their routine antenatal care during the most recent pregnancy?	70(23.3%)
During the most recent pregnancy, did you support use of ANC by your spouse?	251(83.7%)
Did you encourage your wife to attend antenatal clinic?	231(77.0%)
Did you approve your wife going to the ANC clinic?	239(79.7%)
Did you ensure good nutrition for your wife?	273(91.0%)
Did you identify a mode of transportation to the health facility?	223(74.3%)
During the most recent pregnancy, did you discuss about ANC with your wife?	195(65.0%)
Both made decision to attend ANC	118(39.3%)
Who decided the place of delivery?	129(43.0%)
During the most recent pregnancy, did you provide money for clinic costs and medication related to the antenatal clinic visit?	234(78.0%)
During the most recent pregnancy, did you provide money for transport to the clinic?	234(78.0%)
Did you save any money for emergencies related to the pregnancy?	195(65.0%)

The results of bivariate analysis are presented in Table 3. Participants with 1-2 children and 3-4 children were about 4 and 5 times more involved (AOR= 4.57, 95% CI: 1.7, 12.31), p=0.003; AOR= 4.84, 95% CI: 1.59, 14.79, p=0.006) respectively, as compared to those with 5 children and above. Participants who lacked knowledge of the minimum number of ANC visits were less likely to be involved (AOR= 0.37, 95% CI: 0.17, 0.83, p=0.016) compared to those who had that knowledge. The odds of male involvement among respondents whose spouses had unplanned pregnancies was 0.22 times less involved (AOR=0.22, 95% CI: 0.10, 0.48, p<0.001). Participants whose spouses went for ANC during previous pregnancies were about 5 times more involved (AOR= 4.96, 95% CI: 2.37, 10.38, p<0.001) compared to those who did not. Participants who made financial decisions alone were about 0.43 (AOR= 0.42, 95% CI: 0.21, 0.89, p=0.023) less involved as compared to participants who made the decision jointly (Table 4).

**Table 3:** factors associated with male involvement in ANC

Variable	Category	Male Involvement		O.R. (95% C.I.)	Sig.
		No (n=117; 39.0%)	Yes (n=183; 61.0%)		
**Age**	15-24 Years	11(64.7%)	6(35.3%)	0.29(0.09-0.91)	0.034
	25-34 Years	39(38.6%)	62(61.4%)	0.84(0.42-1.69)	0.628
	35-44 Years	49(37.7%)	81(62.3%)	0.88(0.45-1.71)	0.698
	45+	18(34.6%)	34(65.4%)	Ref.	
**Age difference**	Younger than spouse	6(40.0%)	9(60.0%)	0.98(0.33-2.90)	0.964
	Same Age	13(39.4%)	20(60.6%)	1.00(0.46-2.18)	1.000
	1-5 Years Older	43(37.1%)	73(62.9%)	1.10(0.66-1.84)	0.707
	6+ Years older	52(39.4%)	80(60.6%)	Ref.	
**Education Level**	Primary and Below	82(52.2%)	75(47.8%)	0.28(0.14-0.58)	0.001
	Secondary	23(25.0%)	69(75.0%)	0.92(0.41-2.06)	0.845
	Tertiary/ Post-secondary	12(23.5%)	39(76.5%)	Ref.	
**Currently Living with Wife**	Yes	100(37.9%)	164(62.1%)	1.47(0.73-2.95)	0.283
	No	17(47.2%)	19(52.8%)	Ref.	
**Wife’s Employment status**	Formal Employment	2(6.5%)	29(93.5%)	11.73(2.73-50.43)	0.001
	Informal Employment	22(36.1%)	39(63.9%)	1.43(0.79-2.59)	0.231
	Not Employed	93(44.7%)	115(55.3%)	Ref.	
**Level of income**	Below 5,000	51(54.3%)	43(45.7%)	Ref.	
	5,000 - 10,000	46(41.1%)	66(58.9%)	1.70(0.98-2.96)	0.060
	11,000 - 20,000	14(34.1%)	27(65.9%)	2.29(1.07-4.90)	0.33
	Above 21,000	6(11.3%)	47(88.7%)	9.29(3.62-23.82)	<0.001
**Decision on Money earned**	Respondent	63(55.3%)	51(44.7%)	0.28(0.16-0.47)	<0.001
	Wife	19(38.8%)	30(61.2%)	0.54(0.27-1.08)	0.082
	Respondent and wife	35(25.5%)	102(74.5%)	Ref.	
**Number of children**	1-2	69(35.8%)	124(64.2%)	2.93(1.54-5.57)	0.001
	3-4	17(29.8%)	40(70.2%)	3.84(1.72-8.59)	0.001
	5 and Above	31(62.0%)	19(38.0%)	Ref.	
**Knowledge of the minimum required number of ANC Visits**	No	101(50.5%)	99(49.5%)	0.19(0.10-0.34)	<0.001
	Yes	16(16.0%)	84(84.0%)	Ref.	
**Aware of danger signs of pregnancy**	No	100(45.5%)	120(54.5%)	0.32(0.18-0.59)	<0.001
	Yes	17(21.3%)	63(78.8%)	Ref.	
**Pregnancy Planned**	No	56(70.9%)	23(29.1%)	0.16(0.09-0.28)	<0.001
	Yes	61(27.6%)	160(72.4%)	Ref.	
**Went to ANC during previous pregnancies**	Yes	54(27.3%)	144(72.7%)	4.31(2.59-7.15)	<0.001
	No	63(61.8%)	39(38.2%)	Ref.	
**Source of information about ANC**	Mass/Print Media	17(36.2%)	30(63.8%)	3.12(1.26-7.71)	0.014
	Partner/Wife	42(64.6%)	23(35.4%)	0.97(0.41-2.26)	0.942
	Health care Providers	35(23.0%)	117(77.0%)	5.91(2.72-12.87)	<0.001
	Discussion with people	23(63.9%)	13(36.1%)	Ref.	
**Provider Attitude**	Positive	59(33.7%)	116(66.3%)	1.86(0.89-3.87)	0.098
	Negative	41(45.6%)	49(54.4%)	1.13(0.52-2.47)	0.762
	Not applicable	17(48.6%)	18(51.4%)	Ref.	

OR - Crude Odds Ratio; CI-Confidence Interval

**Table 4:** independent predictors of male involvement in ANC

Variable	Category	A.O. R	95% C.I. A.O.R.		Sig.
			Lower	Upper	
Age	18-24 Years	0.22	0.05	1.03	0.055
	25-34 Years	0.39	0.14	1.12	0.080
	35-44 Years	0.43	0.16	1.15	0.092
	45+				
Education Level	Primary and Below	0.80	0.26	2.43	0.692
	Secondary	1.19	0.36	4.00	0.775
	Tertiary/ Post-secondary	Ref.			
Wife’s Employment status	Formal Employment	3.40	0.52	22.01	0.200
	Informal Employment	0.86	0.36	2.07	0.741
	Not Employed	Ref.			
Number of children	1-2	4.57	1.70	12.31	0.003
	3-4	4.84	1.59	14.79	0.006
	5andAbove	Ref.			
Knowledge of the minimum required number of ANC Visits	No	0.37	0.17	0.83	0.016
	Yes	Ref.			
Aware of danger signs of pregnancy	No	0.47	0.20	1.09	0.079
	Yes	Ref.			
Pregnancy Planned	No	0.22	0.10	0.48	<0.001
	Yes	Ref.			
Went to ANC during previous pregnancies	Yes	4.96	2.37	10.38	<0.001
	No	Ref.			
Level of income	Below 5,000	0.55	0.10	2.97	0.484
	5,000 - 10,000	0.49	0.10	2.49	0.388
	11,000 - 20,000	0.65	0.12	3.51	0.617
	21,000 - 30,000	1.39	0.16	12.22	0.766
	Above 30,000	Ref.			
Decision on Money earned	Respondent	0.43	0.21	0.89	0.023
	Wife	0.39	0.15	1.04	0.059
	Respondent and wife	Ref.			
Source of information about ANC	Mass/Print Media	1.58	0.48	5.19	0.454
	Partner/Wife	1.10	0.38	3.17	0.865
	Health care Providers	2.35	0.84	6.52	0.102
	Discussion with people	Ref.			
Provider Attitude	Positive	2.60	0.86	7.87	0.091
	Negative	2.04	0.65	6.37	0.221
	Not applicable	Ref.			

A.O.R-Adjusted Odds Ratio; C.I-Confidence Interval

## Discussion

Findings from this study show that 61% of men supported their spouses to access ANC services. The majority provided nutritional support and the least support was in accompanying their spouses to the clinic. Additionally, almost two-thirds were unaware of the danger signs of pregnancy and also lacked knowledge on the minimum required number of ANC visits. The likelihood of male involvement in ANC was higher among participants with less than four children, and those whose spouses went for ANC during previous pregnancies. These results are consistent with previous studies in western Kenya, Uganda and Tanzania where the proportion of male involvement was 55.8%, 77.8% and 54.4%, respectively [20,24,35]. However, this study reported almost double the levels of male involvement that have been documented in other studies in Nairobi, Kenya (26.2%), Ethiopia (29.8%), and Ghana (35%) [21,23,36].

Participants who had less than 4 children were more likely to be involved compared to those with 5 or more children. While this is in line with findings from similar studies [36,37], these studies made comparison to men who lacked children unlike the present study. In contrast, a study in Tanzania [38] found that having more than 4 children was significantly associated with male involvement in maternity care, and was attributed to fertility preferences, concern over mothers´ health, and familiarity with the health system. It is plausible that a positive clinic experience and interest in the health of the mother and baby could have influenced the involvement of men with less than 4 children. However, men with more than 5 children could have been less involved due to socio-cultural beliefs that ANC is a women´s affair or previous negative experiences with the health system as elucidated by other studies in the region [22,35,39,40].

The findings further show that men whose spouses went for ANC during previous pregnancies were more likely to be involved compared to those who did not. These findings suggest that men appreciated the need to support their wives based on counseling and experience from previous ANC interactions [29], but it is also likely that they had a positive experience with the health system that motivated them [41]. An interesting finding was that men who reported unplanned pregnancies were less likely to be involved compared to planned pregnancies. This finding suggests that men portray a greater sense of responsibility when a pregnancy is accepted by the couple as affirmed by a study in Kenya [29]. However, this is contrasted by a study in Uganda where the likelihood of involvement was higher for unplanned pregnancies [22].

While level of education was not significant in contrast to a number of studies [22,29,36,37], men who lacked knowledge on the minimum number of ANC visits were less likely to be involved. These findings suggest that an understanding of the risks, benefits and roles that men can play could be an important factor in their involvement, and is consistent with existing studies which found a significant association between knowledge of ANC and male involvement [37,42-44]. In addition, participants who made financial decisions individually, were less likely to be involved compared to those who made joint decisions with their spouses. It is plausible to state that joint decision-making meant spouses had good communication that made it easier to discuss and prioritize ANC. This finding is consistent with studies which have reported that positive interpersonal relationships lead to equitable decision-making [45], and conversely poor communication contributes to lack of male involvement [39,46].

**Study limitations:** this study had several limitations. First, this study only interviewed male respondents and did not interview their spouses to corroborate the information reported. There is a likelihood of social desirability bias from some of the respondents who may have wanted to appear more involved than they actually had been. Secondly, the study is likely to have suffered from recall limitation since we sampled participants whose spouses had given birth twelve months prior to the study, and they may have forgotten details of their experiences. Finally, Kitui East sub-County is geographically sparse with part of the sub-County peri-urban and densely populated, while a larger section was rural and sparsely populated. While we attempted to account for this through purposively selecting the 3 wards where data were collected, there is a likelihood that these differentials may not be accurately accounted for in the results. Despite these limitations, this study provides empirical evidence on the levels of male partner involvement and associated factors in Kitui East sub-county, Kenya.

## Conclusion

This study has demonstrated that more than half of the participants reported involvement in ANC services. The majority of the participants provided nutrition support, while fewer participants accompanied their spouses to the clinic. Fewer children and previous ANC experience were significantly associated with male involvement. On the other hand, unplanned pregnancies, lack of knowledge on ANC and individual decision-making on finances were least associated with male involvement. Therefore, empowering men with knowledge of ANC and enhancing couple communication are critical strategies to enhance male involvement in ANC services.

### What is known about this topic

- Male partner involvement in ANC results in improved maternal outcomes;
- Previous studies have documented the levels, barriers and effect of male partner involvement on utilization of ANC services;
- Studies have also documented individual domains of male partner involvement such as financial, physical and decision-making support, and associated factors.

### What this study adds

- An understanding of the level of male partner involvement in ANC in Kitui East sub-County;
- Empirical evidence on how the four main domains of male involvement (physical, financial, psycho-social and decision-making support) interact to influence male involvement in ANC, and the associated factors.
